# A Meta-Analysis of Preclinical Studies to Investigate the Effect of *Panax ginseng* on Alcohol-Associated Liver Disease

**DOI:** 10.3390/antiox12040841

**Published:** 2023-03-31

**Authors:** Keungmo Yang, Tom Ryu, Beom Sun Chung

**Affiliations:** 1 Department of Internal Medicine, Division of Gastroenterology and Hepatology, College of Medicine, The Catholic University of Korea, Seoul 06591, Republic of Korea; 2 Department of Internal Medicine, Institute for Digestive Research, Digestive Disease Center, College of Medicine, Soonchunhyang University, Seoul 04401, Republic of Korea; 3 Department of Anatomy, College of Medicine, Yonsei University Wonju, Wonju 26426, Republic of Korea

**Keywords:** meta-analysis, *Panax ginseng*, alcohol-associated liver disease, inflammatory response, anti-inflammation, antioxidant

## Abstract

Alcohol-associated liver disease (ALD) has become a major global concern, but the development of effective drugs remains a challenge despite numerous preclinical and clinical pieces of research on the effects of natural compounds. To address this, a meta-analysis was conducted on the efficacy of *Panax ginseng* for ALD based on preclinical studies. We identified 18 relevant studies from PubMed, Web of Science, and Cochrane Library database and evaluated their methodological quality using the Systematic Review Centre for Laboratory animal Experimentation tool. We analyzed the data using *I*^2^, *p*-values, and fixed effects models to assess overall efficacy and heterogeneity. The results of the meta-analysis suggested that *Panax ginseng* treatment is effective in reducing the levels of inflammatory markers associated with hepatic injury caused by ALD in animal experiments. Additionally, the administration of *Panax ginseng* was found to down-regulate inflammatory cytokines and attenuate lipid metabolism in ALD. Moreover, *Panax ginseng* markedly improved the antioxidant systems in ALD. Therefore, we concluded that *Panax ginseng* has the potential to be a promising therapeutic agent for ALD. Further research is needed to confirm these findings and to determine the optimal dosage and duration of treatment for patients with ALD.

## 1. Introduction

Adverse events resulting from excessive alcohol use have increased worldwide [1]. The percentage of death related to alcohol use in Europe was 10.1%, and digestive diseases, including liver disease, were reported as 8.3 deaths per 100,000 people [2]. Over the past decade, the rate of monthly alcohol consumption has increased, and the incidence of alcohol-associated diseases (ALD) has also increased [3].

Manifestations of ALD include fatty liver, liver inflammation, hepatic fibrosis, cirrhosis, and hepatocellular carcinoma (HCC). In hepatocytes, alcohol is converted into acetaldehyde by alcohol dehydrogenase, and chronic alcohol drinking induces cytochrome P450 2E1 (CYP2E1), which can also metabolize alcohol to acetaldehyde. With excessive alcohol consumption, inflammation causes reactive oxygen species (ROS) production, and it is generated as a byproduct of CYP2E1. ROS stimulate lipid peroxidation, alter immune reactions, and activate hepatic stellate cells [4]. Alcoholic fatty liver is caused by the upregulation of sterol regulatory element-binding protein 1c (SREBP-1c) and an altered ratio of reduced nicotinamide adenine dinucleotide (NAD) and oxidized NAD [5,6]. Hepatic inflammation is mainly induced by pathogen-associated molecular patterns or damage-associated molecular patterns, and minor factors include microRNAs, mitochondrial double-stranded RNA (mtdsRNA), and other metabolites [7]. Moreover, activated hepatic stellate cells induced by acetaldehyde production stimulate extracellular matrix production, resulting in liver fibrosis, and advanced fibrosis becomes cirrhosis, which narrows the sinusoid and disrupts blood flow [8,9]. Finally, liver inflammation and oxidative stress induced by alcohol cause DNA damage to hepatocytes and contribute to the development of HCC [10]. 

Various studies have shown that nutritional supplements and medical interventions can improve the survival of ALD. Daily intake of 35 kcal/kg and protein at 1.2 to 1.5 g/kg, including nighttime snacks, is suggested for patients with ALD [11]. In cases of severe ALD, as assessed by Maddrey’s discriminant-function score ≥ 32, prednisolone was associated with a reduction of 28-day mortality, but there was no improvement in outcomes at 90 days or longer [12]. Treatment with pentoxifylline only or a combination of pentoxifylline and prednisolone did not show a benefit in survival rate [12,13]. Anti-tumor necrosis factor (anti-TNF) agents, including Infliximab and Etanercept, did not show better survival rates in severe alcoholic hepatitis compared to a placebo [14,15]. A study that evaluated N-acetylcysteine treatment plus prednisolone with a comparison to prednisolone only showed a decrease in 1-month mortality with combination therapy compared to monotherapy with prednisolone, but not in 6 months [16]. There are ethical controversies regarding liver transplantation in alcoholic liver disease. The main problem is that ALD is a self-inflicted disease, and the other controversy is the risk of recidivism [17]. Nevertheless, an early liver transplant is known to be effective in the treatment of patients with no response to steroids [18].

Interestingly, natural compounds are considered potential therapeutic options for ALD. Curcumin regulates the NF-E2-related factor 2 (Nrf2)–farnesoid X receptor (FXR) pathway and has been shown to improve hepatic steatosis [19]. Anthocyanin treatment reduced the expression of Toll-like receptor 4 (TLR4) and TNF-alpha in a study on ALD mice [20]. Moreover, the citrus narirutin fraction was found to suppress pro-inflammatory cytokines in a mouse model of ALD [21]. However, apart from the cessation of alcohol drinking, there is currently no definitive treatment option to reverse the progression of ALD with additional treatment.

Given these points of view, *Panax ginseng* has emerged as one of the potential treatment options for ALD patients. *Panax ginseng*, also known as Korean ginseng, is one of the most commonly used ginsengs [22]. Ginsenoside, the main active component of *Panax ginseng*, has numerous pharmacological effects such as antioxidant, anti-inflammatory, and anticancer effects [23]. In addition to its anti-inflammatory and antioxidative effects, *Panax ginseng* is known to play key roles in anti-fibrosis in various organs, such as the liver, lung, heart, and kidney [24]. 

Recent studies have focused on the effects of ginsenoside on ALD, which involves the pathologic mechanism of inflammation and oxidative stress. One recent study showed that ginsenoside Rg1 could improve ALD through the TLR/nuclear factor kappa B (NF-kB) pathway, while another study concluded that ginsenoside F2 treatment attenuated ALD by increasing IL-10 expression and decreasing interleukin-17 expression and Th17 cells [25,26]. With these regards, a well-organized systematic review and meta-analysis demonstrating the efficacy of *Panax ginseng* in ALD could provide established evidence for future trials.

Here, we conducted a meta-analysis using preclinical studies of ALD and a systematic review focused on hepatic inflammation, liver lipogenesis, and oxidative stress during acute and chronic alcohol consumption. We aim to investigate the effects of *Panax ginseng* on serum markers of hepatic inflammation and steatosis and to also focus on the inflammatory cytokines and antioxidant system during the treatment of ALD with *Panax ginseng*.

## 2. Materials and Methods

### 2.1. Literature Searching

The current meta-analysis, following the preferred reporting items for systematic reviews and meta-analyses (PRISMA) guidelines, was conducted by 2 independent researchers (K.Y. and T.R.) and registered in the prospective international register of systematic reviews (PROSPERO) under registration number CRD42023389133 [27]. To comprehensively search for reliable literature on the therapeutic efficacy of *Panax ginseng* for ALD, 3 databases (PubMed, Web of Science, and the Cochrane Library) were utilized from the inception to February 2023. The main keywords used in the search were “Ginseng” and “Alcoholic liver disease”, and their variations were additionally searched. A detailed search strategy is provided in Table 1.

### 2.2. Criteria for Inclusion and Exclusion

The systematic review included literature based on the ALD model in animals that met the following inclusion criteria: (1) at least 1 serological marker reflecting the severity of ALD was included; (2) experimental (with *Panax ginseng* treatment) and control (without *Panax ginseng* treatment) groups should be clearly divided in ALD-induced rodents; (3) the literature was written in English; (4) all animal experiments were approved by the Institutional Animal Care and Use Committee (IACUC). 

The following criteria were used to exclude studies from the final analysis: (1) duplicate literature identified from different databases; (2) studies not related to the therapeutic efficacy of *Panax ginseng* in ALD; (3) studies using natural products other than *Panax ginseng*; (4) studies without animal experiments (only in vitro experiments); (5) any review articles or clinical trials; (6) insufficient data on primary outcomes; (8) conference abstracts, books, or theses.

### 2.3. Data Extraction

Two independent researchers extracted primary data from the included studies. The extracted information included the name of the first author and the publication year, the country of the authors, sample size in each group, animal species, diet model of ALD, detailed components of *Panax ginseng*, and treatment dose, route, and times. For data presented in graphs, digitizing software was used for extraction. If there were any differences in the data extraction between the 2 initial researchers, they were resolved through discussion with a third reviewer.

### 2.4. Quality Assessment

The assessment of study quality was done using the risk of bias tool outlined in the SYstematic Review Centre for Laboratory animal Experimentation (SYRCLE) guideline for animal studies [28]. The quality investigation is divided into the following categories: (1) Sequence generation; (2) Baseline characteristics; (3) Allocation concealment; (4) Random housing; (5) Blinding for the performance bias; (6) Random outcome assessment; (7) Blinding for the detection bias; (8) Incomplete outcome data; (9) Selective outcome data; (10) Other sources of bias. The evaluation of each category was categorized as having a high, low, or uncertain risk of bias.

### 2.5. Statistical Analysis

To investigate the effectiveness of *Panax ginseng* treatment in ALD, a meta-analysis was conducted using both ReviewManager (RevMan 5.4) and R software (version 4.2.1; R Foundation, Inc; http://cran.r-project.org (accessed on 21 February 2023)). The standardized mean difference (SMD) with a 95% confidence interval (CI) was calculated. To evaluate heterogeneity among the included studies, the *I*^2^ statistic and Cochranes’ Q-square test were utilized. For minor heterogeneity (*I*^2^ ≤ 50% or *p* ≥ 0.1), a fixed-effects model was used, while a random-effects model was used for major heterogeneity *(I*^2^ > 50% or *p* < 0.1).

## 3. Results

### 3.1. Identification and Selection of Study

**Figure 1:**
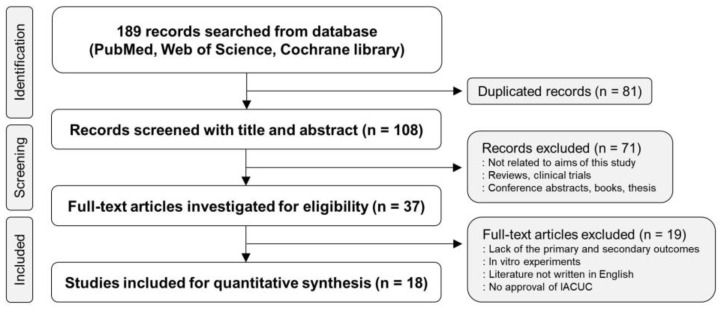
The flow diagram for literature searching.

We followed the literature search strategy outlined in Figure 1 to identify articles for the present meta-analysis. Initially, we searched three databases (PubMed, Web of Science, and Cochrane Library) and found a total of 189 records, out of which 81 duplicates were removed after the first screening. We then evaluated the titles and abstracts of the remaining records and excluded those that were not relevant to the efficacy of Panax ginseng in ALD, systematic reviews, clinical trials, conference abstracts, books, and theses. After this screening process, we carefully reviewed 37 full-text articles for eligibility and ultimately included 18 studies in our meta-analysis (Figure 1) [25,26,29,30,31,32,33,34,35,36,37,38,39,40,41,42,43,44].

### 3.2. Study Characteristics

Table 2 summarizes the main features of the experiments involved in the systematic review and meta-analysis. The 18 articles included in the study were published between 2011 and 2022, and the majority of the authors were Korean (33.3%) or Chinese (66.7%). Mice (77.8%) and rats (22.2%) were the animal species used, and various animal models were selected to induce ALD in rodents, such as single binge, chronic feeding, or chronic feeding plus binge. The treatment agents used in the studies ranged from single components such as ginsenoside Rg1 or F2 to Panax ginseng itself. The drugs were mainly administered orally (Table 2 and Table S1).

### 3.3. Quality Assessment

**Figure 2:**
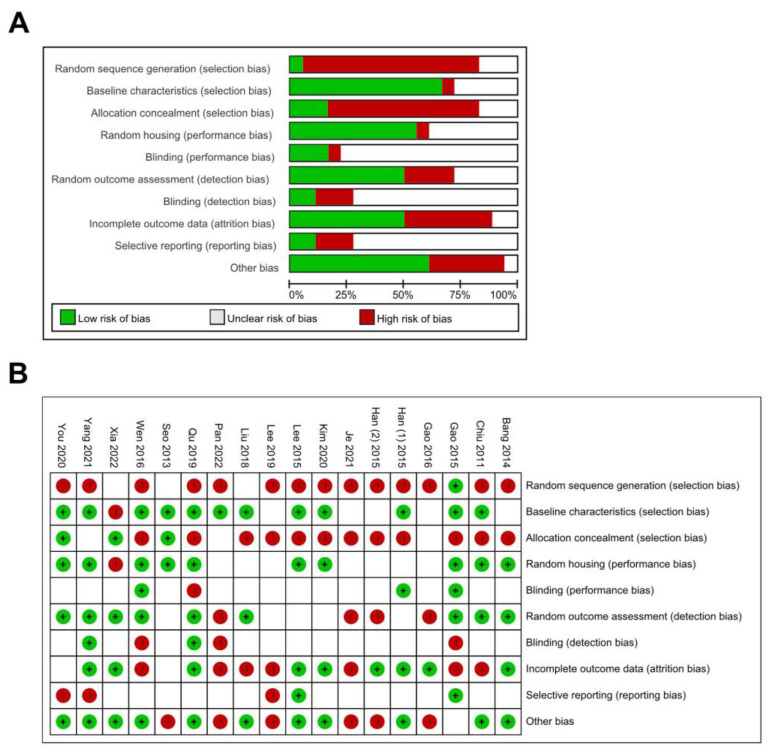
Quality evaluation of included studies [25,26,29,30,31,32,33,34,35,36,37,38,39,40,41,42,43,44] according to the SYRCLE’s risk of bias tool. (**A**) Risk of bias graph. (**B**) Risk of bias summary with included studies.

The SYRCLE criteria for animal studies were used to evaluate the quality of the articles that were included in the analysis (Figure 2A). Out of the 18 studies that were analyzed, only one piece of literature had a low risk of bias in terms of random sequence generation. For baseline characteristics, 12 studies had a low risk of bias, while one or five studies had a high or unclear risk of bias. While 12 included articles had a high risk of selection bias due to allocation concealment, 10 studies had a low risk of performance bias due to random housing. Estimating the risk of blinding and reporting bias was challenging due to the nature of in vivo experiments. The risk of bias assessment for each study was summarized in Figure 2B.

### 3.4. The Effects of Panax Ginseng on Hepatic Inflammation in ALD

#### 3.4.1. Inflammatory Markers in Alcohol-Associated Liver Injury

**Figure 3:**
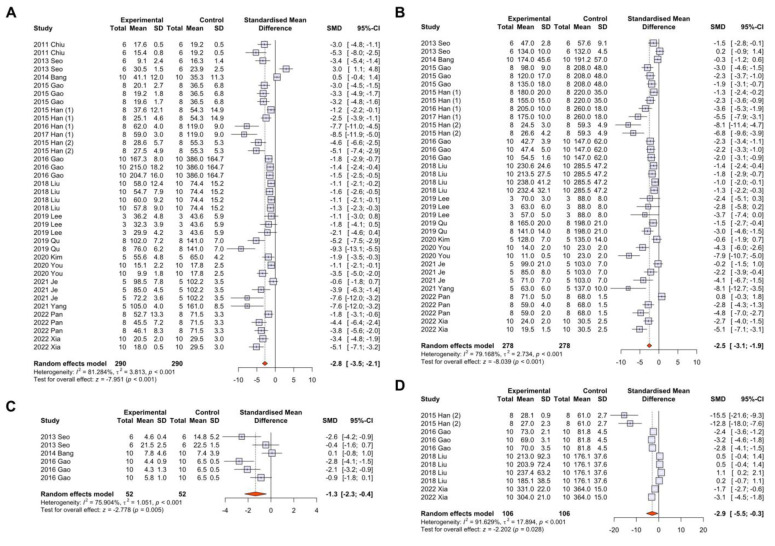
The effect of Panax ginseng on serological inflammatory markers in [25,29,30,31,32,33,34,38,39,40,41,42,43,44]. Forest plots for comparison (**A**) Serum alanine aminotransferase (ALT) levels. (**B**) Serum aspartate aminotransferase (AST) levels. (**C**) Serum gamma-glutamyltransferase (GGT) levels. (**D**) Serum alkaline phosphatase (ALP) levels.

Inflammatory liver injury is a critical event in the progression of ALD caused by persistent alcohol consumption. The major aim of this meta-analysis was to investigate the effects of Panax ginseng on serological markers of inflammatory liver injury in animal models of ALD. A total of 16 studies (with 580 rodents) were analyzed to determine the effects of Panax ginseng on alanine aminotransferase (ALT) levels (Figure 3A). Due to the high heterogeneity between experimental and control groups, the random-effects model was selected for further investigation. The results showed a significant decrease in ALT levels in animals treated with Panax ginseng (SMD: −2.8 U/L; 95% CI: −3.5–−2.1 U/L; *p*-value < 0.001; Figure 3A). For the effects of Panax ginseng on aspartate aminotransferase (AST) levels, 15 studies (with 556 rodents) were analyzed, and similar to the analysis of ALT, a significant decrease in AST levels was observed in ALD animals treated with Panax ginseng (SMD: −2.5 U/L; 95% CI: −3.1–−1.9 U/L; *p*-value < 0.001; Figure 3B). In the aspect of serum gamma-glutamyltransferase (GGT) and alkaline phosphatase (ALP) levels, Panax ginseng notably presented beneficial effects (Figure 3C,D). These findings indicate a protective effect of Panax ginseng in ALD-induced hepatic inflammation in preclinical animal models.

#### 3.4.2. Subgroup Analysis According to the Animal Models of Alcohol-Associated Liver Disease

**Figure 4:**
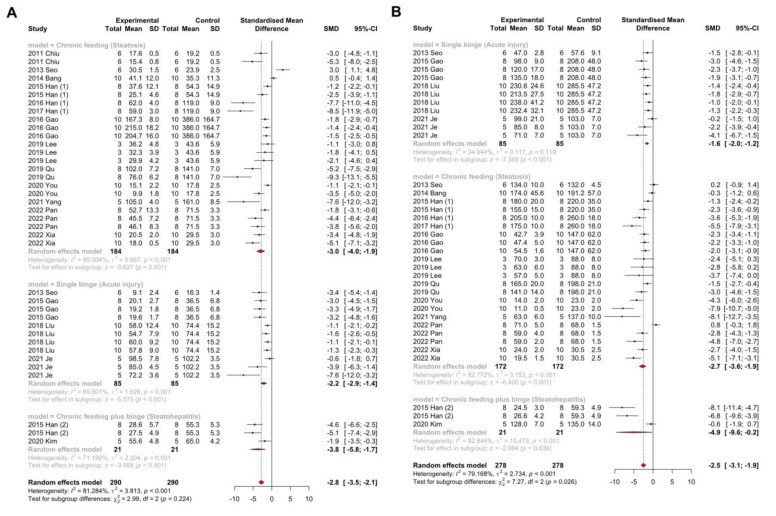
Subgroup analysis according to the animal models of alcohol-associated liver disease in [25,26,29,30,31,32,33,36,38,39,40,41,42,43,44]. Forest plots for comparison (**A**) Serum alanine aminotransferase (ALT) levels. (**B**) Serum aspartate aminotransferase (AST) levels.

In preclinical studies of ALD, various animal models are utilized to induce different stages of ALD. Generally, binge drinking induces inflammation (acute injury), chronic feeding induces steatosis, and a combination of binge and chronic feeding leads to steatohepatitis. Therefore, we conducted a subgroup analysis according to animal models to evaluate the effects of Panax ginseng on ALT and AST. Interestingly, regardless of the animal model, both ALT (*p*-value in steatosis < 0.001; *p*-value in acute injury < 0.001; *p*-value in steatohepatitis < 0.001) and AST (*p*-value in steatosis < 0.001; *p*-value in acute injury < 0.001; *p*-value in steatohepatitis = 0.039) levels showed significant anti-inflammatory effects in the group treated with Panax ginseng (Figure 4A,B). Based on these results, we concluded that the administration of Panax ginseng affects both inflammation and steatosis in ALD.

#### 3.4.3. The Effects of Panax Ginseng on Lactate Dehydrogenase

**Figure 5:**
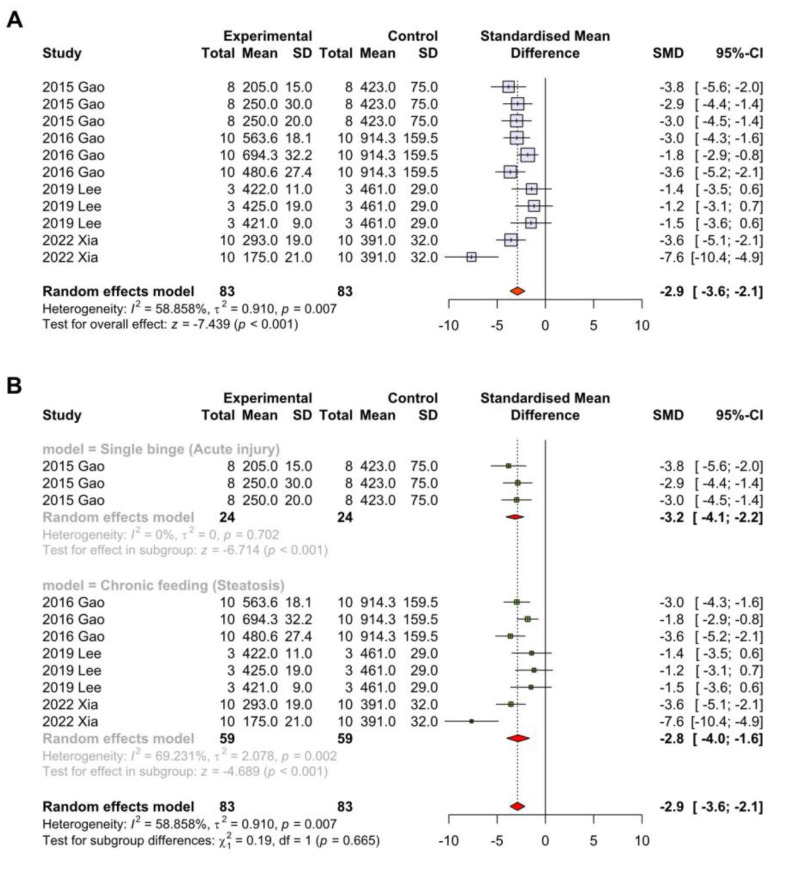
The effect of Panax ginseng on lactate dehydrogenase (LDH) in [25,32,36,39]. Forest plots for comparison (**A**) Total groups. (**B**) Subgroup analysis according to the animal models.

Lactate dehydrogenase (LDH) is an enzyme involved in anaerobic metabolism that is commonly used as a marker of tissue damage. LDH levels are often elevated due to liver injury and cell death in ALD. Elevated LDH levels have been associated with increased severity of ALD and can be used as a prognostic indicator for disease progression [4,11]. We analyzed a total of 4 studies with 166 animals to evaluate the impact of Panax ginseng on serum LDH levels, as shown in Figure 5A. The high heterogeneity between the experimental and control groups led us to use the random-effects model for further investigation. The results revealed a significant reduction in LDH levels in animals treated with Panax ginseng (SMD: −2.9 mg/dL; 95% CI: −3.6–−2.1 mg/dL; *p*-value < 0.001; Figure 5A). In subgroup analysis based on the ALD models in animals, Panax ginseng treatment significantly reduced LDH levels in both acute injury (*p*-value < 0.001) and steatosis (*p*-value < 0.001; Figure 5B). Based on these results, we demonstrated that Panax ginseng could improve overall tissue damage caused by alcohol.

### 3.5. The Effects of Panax Ginseng on Lipid Metabolism in ALD

#### 3.5.1. Systemic Markers of Lipid Metabolism and Cholestasis in Alcohol-Associated Liver Injury

**Figure 6:**
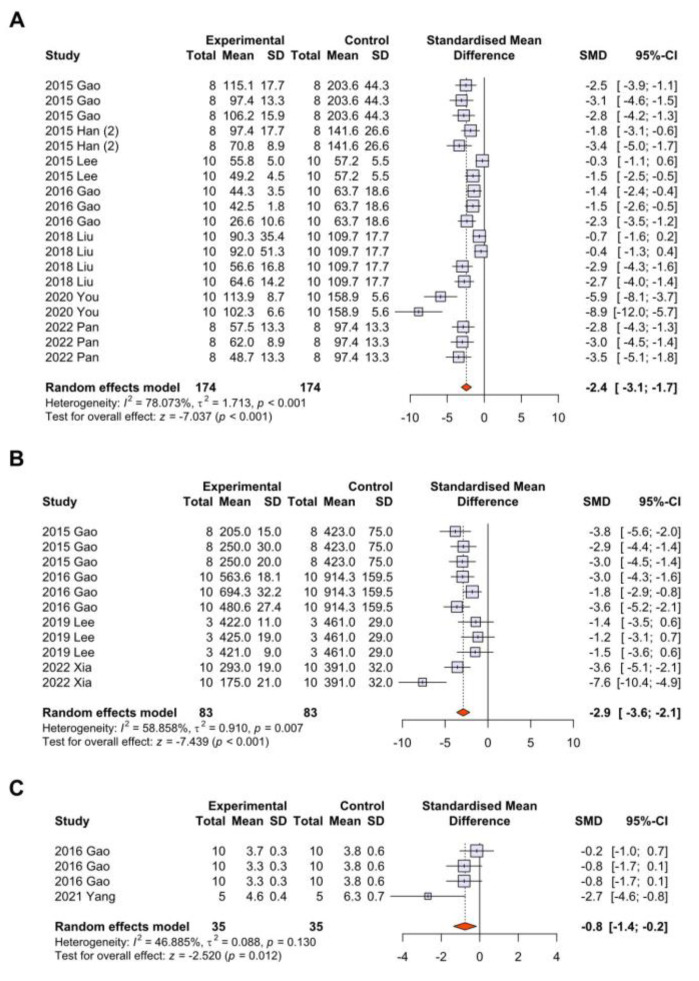
The effect of Panax ginseng on systemic lipid metabolism and cholestasis in [25,32,34,35,36,38,39,41,43,44]. Forest plots for comparison (**A**) Serum triglyceride (TG) levels. (**B**) Serum low-density lipoprotein (LDL) levels. (**C**) Serum total bilirubin levels.

**Figure 7:**
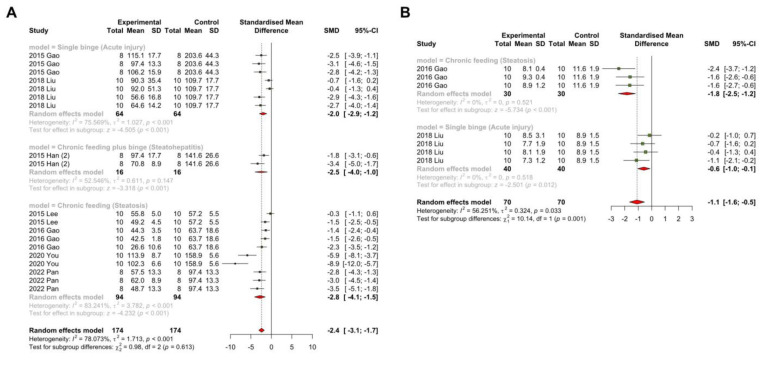
Subgroup analysis according to the animal models of alcohol-associated liver disease in [32,34,35,36,38,41,44]. Forest plots for comparison (**A**) Serum triglyceride (TG) levels. (**B**) Serum low-density lipoprotein (LDL) levels.

The ‘multiple hit’ hypothesis proposes that persistent alcohol consumption leads to hyperlipidemia and steatosis and initiates the pathophysiology of ALD progression [4,45]. To investigate whether Panax ginseng administration affects lipid metabolism in ALD-induced animals, we analyzed seven studies with a total of 348 animals and found a significant decrease in serum triglyceride (TG; SMD: −2.4 mg/dL; 95% CI: −3.1–−1.7 mg/dL; *p*-value < 0.001) and low-density lipoprotein (LDL; SMD: −2.9 mg/dL; 95% CI: −3.6–−2.1 mg/dL; *p*-value < 0.001) levels after Panax ginseng treatment (Figure 6A,B). In the subgroup analysis of lipid metabolism markers according to different animal models, Panax ginseng treatment significantly attenuated serological TG (*p*-value in steatosis < 0.001; *p*-value in acute injury < 0.001; *p*-value in steatohepatitis < 0.001) and LDL (*p*-value in steatosis < 0.001; *p*-value in acute injury = 0.012) levels in all ALD models (Figure 7A,B). These results indicate that Panax ginseng plays a protective role not only in inflammation but also in lipid metabolism in the onset of ALD. Furthermore, we evaluated the changes in serum total bilirubin levels to investigate the efficacy of Panax ginseng on cholestasis in ALD. Interestingly, similar to markers of inflammation or lipid metabolism, the total bilirubin levels were significantly improved in Panax ginseng-treated group (SMD: −0.8 μmol/L; 95% CI: −1.4–−0.2 μmol/L; *p*-value = 0.012; Figure 6C).

#### 3.5.2. The Effect of Panax Ginseng on the Hepatic Lipid Metabolism

**Figure 8:**
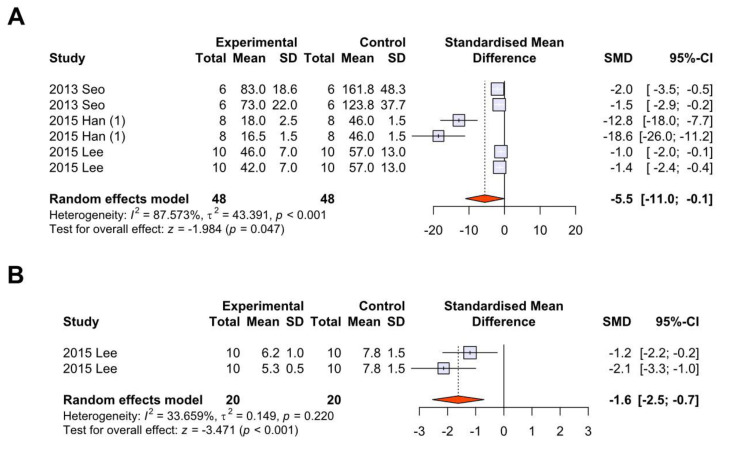
The effect of Panax ginseng on the hepatic lipid metabolism in [30,33,35]. Forest plots for comparison (**A**) Hepatic triglyceride (TG) levels. (**B**) Hepatic total cholesterol (TC) levels.

We investigated whether Panax ginseng treatment has an impact on hepatic lipid metabolism in the development of ALD. To do this, we examined the overall effects of Panax ginseng on markers related to hepatic lipid metabolism in animals with ALD. Our analysis revealed a significant reduction in hepatic TG levels in ALD-induced animals treated with Panax ginseng (SMD: −5.5 mg/g; 95% CI: −11.0–−0.1 mg/g; *p*-value = 0.047), as shown in Figure 8A. To further investigate the impact of Panax ginseng on hepatic lipid accumulation, we assessed the changes in hepatic TC levels. Consistent with the decrease in hepatic TG levels, we observed a significant reduction in hepatic TC levels with Panax ginseng treatment (Figure 8B). 

### 3.6. The Effects of Panax Ginseng on Inflammatory Cytokines in ALD

**Figure 9:**
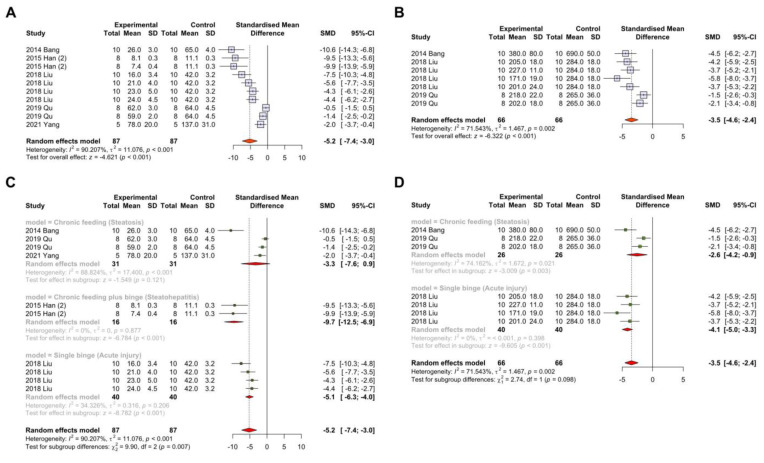
The effect of Panax ginseng on the inflammatory cytokines in [31,34,38,40,43]. Forest plots for comparison (**A**) Interleukin-1 beta (Il-1β). (**B**) Tumor necrosis factor (TNF). (**C**) Subgroup analysis of Il-1β. (**D**) Subgroup analysis of TNF.

In the pathophysiology of ALD, inflammatory cytokines play a critical role in the progression of the disease. Among various pro-inflammatory cytokines, interleukin-1 beta (Il-1β) and tumor necrosis factor (TNF) are the most important in alcohol-induced hepatic inflammation [4]. Therefore, we investigated whether Panax ginseng affects the expression of these inflammatory cytokines. Interestingly, both Il-1β (SMD: −5.2 pg/mL; 95% CI: −7.4 to −3.0 pg/mL; *p*-value < 0.001) and TNF (SMD: −3.5 pg/mL; 95% CI: −4.6 to −2.4 pg/mL; *p*-value < 0.001) expression were significantly suppressed in the group treated with Panax ginseng (Figure 8A,B). Furthermore, regardless of animal models such as acute inflammation, steatosis, and steatohepatitis, Panax ginseng showed beneficial results in ALD (Figure 8C,D). These results suggest that the inhibition of inflammatory cytokines is related to the protective mechanism of Panax ginseng in ALD (Figure 9).

### 3.7. The Effects of Panax Ginseng on Oxidative Stress and Antioxidant System in ALD

**Figure 10:**
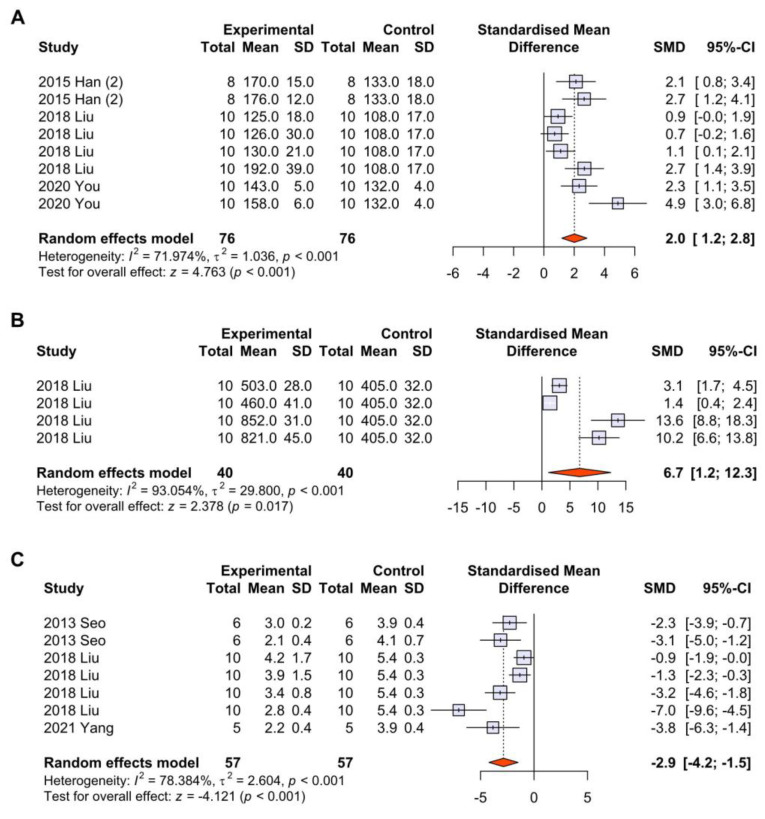
The effect of Panax ginseng on the antioxidant system in alcohol-associated liver disease in [30,34,38,41,43]. Forest plots for comparison (**A**) Superoxide dismutase (SOD) levels. (**B**) Glutathione (GSH) levels. (**C**) Malondialdehyde (MDA) levels.

**Figure 11:**
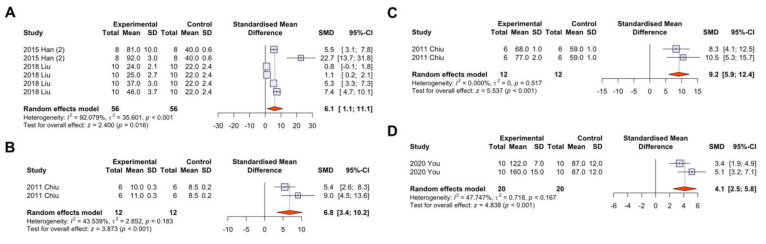
The effect of Panax ginseng on the other factors antioxidant system in the liver in [29,34,38,41]. Forest plots for comparison (**A**) Glutathione peroxidase (GPx) levels. (**B**) Glutathione reductase (GR) levels. (**C**) Glutathione S-transferases (GST) levels. (**D**) Catalase (CAT) levels.

Alcohol disturbs various antioxidant systems present in the liver, and the increased intrahepatic oxidative stress from alcohol promotes the progression of ALD [4,7]. Therefore, we investigated whether Panax ginseng has a positive effect on these antioxidant factors such as superoxide dismutase (SOD; SMD: +2.0 mU/mg; 95% CI: +1.2–+2.8 mU/mg; *p*-value < 0.001) and glutathione (GSH; SMD: +6.7 nmol/mg; 95% CI: +1.2–+12.3 nmol/mg; *p*-value < 0.001) in ALD-induced animals. Remarkably, after Panax ginseng treatment, there was a significant increase in SOD and GSH levels (Figure 10A,B), while malondialdehyde (MDA; SMD: −2.9 nmol/L; 95% CI: −4.2–−1.5 nmol/L; *p*-value < 0.001) levels prominently decreased (Figure 10C). After investigating other factors involved in the hepatic antioxidant system, we found that the levels of glutathione peroxidase (GPx), glutathione S-transferases (GST), and catalase (CAT) were significantly increased in animals treated with Panax ginseng, providing multiple lines of evidence that the protective effect of Panax ginseng in ALD is associated with the activation of the antioxidant system (Figure 11).

## 4. Discussion

The pathophysiology of ALD includes lipogenesis, inflammation, and additional fibrosis and cirrhosis. Hepatic ethanol oxidation induces the translocation of SREBP-1c from the endoplasmic reticulum to the Golgi apparatus, producing a transcriptionally active SREBP fragment that enhances the expression of genes related to lipogenesis, such as fatty acid synthase and malic enzyme [46]. Moreover, acute and chronic alcohol consumption can result in inefficient fatty acid import and alter very low-density lipoprotein secretion [47,48]. The ‘second hit,’ such as an additional toxic insult or nutritional defect, can convert hepatic steatosis to steatohepatitis, which occurs in about 15–20 percent of patients with fatty liver disease [45,49]. In the inflammation phase, both proinflammatory cells, such as Kupffer cells and infiltrating inflammatory cells, such as neutrophils and lymphocytes, can cause damage to normal tissue by increasing abnormal phagocytic activities and cytokine production (e.g., TNF and interleukin-1) [50,51]. In addition, metabolized acetaldehyde can change the mitochondrial structure and decrease adenosine triphosphate generation, thereby producing ROS, which mediates protein adducts and neoantigens in the inflammatory response [4,52]. Despite these pathologic mechanisms regarding ALD, there is a lack of recent study arrangements or reviews targeting alcohol-induced steatosis and inflammation, except for the effect of microbial treatment on the disease, published in 2022 [53].

On the other hand, the multiple effects of *Panax ginseng* on liver protection are well-known in terms of antioxidation and anti-inflammation. The extract of *Panax ginseng* enhances the activities of self-antioxidant enzymes such as CAT, SOD, GPx, and glutathione reductase (GR) in the liver of aged rats [54]. A study revealed the molecular mechanism of hepatoprotection of *Panax ginseng* by suppressing the expression of inducible nitric oxide synthase (iNOS) protein [55]. *Panax ginseng* was also shown to inhibit TNF-α-stimulated NF-kB and suppress the secretion of Il-1β in carbon tetrachloride-treated mice [56]. Furthermore, ginsenoside Rg3 and Rg2 are major components of *Panax ginseng* and are proven to have a beneficial effect on the human body [57]. Ginsenoside Rg3 was found to have a suppressive effect on liver cancer cell lines and also on in vivo hepatocellular tumor growth by inducing apoptosis [58,59]. Additionally, ginsenoside Rg3 has been shown to restore hepatitis C virus (HCV)-induced dynamin-related protein 1-mediated mitochondrial function, thereby suppressing HCV infection [60]. Ginsenoside Rg2 was implicated to have a beneficial effect on the neurological system against glutamate-induced apoptosis and oxidative stress, and the further effect was proven to be one of the potential therapeutic options in Alzheimer’s disease [61]. Also, other ginsenosides, such as Rb1, Rg1, Rd, Re, Ro, and F2, were known to have various beneficial effects on liver health conditions [26,56].

In this systematic review and meta-analysis study, we investigated various components of *Panax ginseng* (e.g., Rg1, F2, Rc, and Korean red ginseng extract) from different studies. Each study used acute, chronic, and acute-on-chronic alcohol drinking models to mimic ALD in humans for mice experiments. This manuscript presents data using Forest plots and investigates serum inflammatory markers such as ALT, AST, GGT, and ALP. Although GGT and ALP are also cholestasis markers for biliary problems, they could be meaningful for alcoholic exposure in the pathological condition of the liver [62,63]. Moreover, we explored the data regarding serum ALT and AST with subgroup analysis by dividing groups into chronic feeding (steatosis), single binge (acute injury), and chronic feeding plus binge (steatohepatitis) to provide similarity to the human ALD subgroups. Interestingly, our data investigation revealed that *Panax ginseng* had a significantly beneficial effect on liver inflammation, even with all subgroup analyses. Also, we analyzed pro-inflammatory cytokines from the liver, such as Il-1β and TNF, with the total group and subgroup analysis finding the significant effectiveness of ginseng in suppressing the release of these cytokines. In addition to inflammation itself, we analyzed the effect of *Panax ginseng* on preventing cell lysis or apoptosis using serum lactate LDH. As a result, both acute and chronic models of ALD showed a beneficial effect of *Panax ginseng* in terms of cell damage. We investigated not only inflammation but also alcohol-associated steatosis by utilizing serum TG, serum LDL, hepatic TG, and hepatic cholesterol levels. Our data showed a further helpful influence of *Panax ginseng* for the prevention or treatment of alcohol-associated steatosis.

On the other hand, we tried to reveal the effect of *Panax ginseng* on antioxidative influence on ALD. Several important antioxidants, including GSH, SOD, GPx, CAT, and GR, have a significant correlation with the suppression of cancer development, obesity, and inflammatory diseases [64,65]. Treatment of *Panax ginseng* components for the ALD model of rodents resulted in a prominent activation of hepatic antioxidant systems in this meta-analysis. Furthermore, MDA is known to be a product of polyunsaturated fatty acid peroxidation, and several methods are used to assess and quantify the level of oxidative stress [66]. This study unveiled the significant decrease of MDA with the intervention of *Panax ginseng* in alcohol-induced liver damage, suggesting the suppressive effect of *Panax ginseng* on oxidative stress in ALD. 

Although this study revealed numerous protective aspects of *Panax ginseng* on ALD with several pathophysiologies, there are some limitations. First, we have not considered the inter-organ axis, which is activated upon alcohol exposure. The gut-liver axis, which is related to portal vein connection, bacterial translocation, and leaky gut, would be an important factor in future studies. Moreover, the adipose tissue-liver axis, which results in the production of damage-associated molecular patterns, could also be one of the crucial factors in ALD development. Second, due to the nature of a systematic review and meta-analysis study, the structure-activity or detailed molecular mechanism of *Panax ginseng* have not been elucidated. Third, we only utilized the previous studies regarding preclinical research using rodents. Further investigation using human samples demonstrating the correlation between *Panax ginseng* administration and suppression of ALD would be necessary.

## 5. Conclusions

In this systematic review and meta-analysis study, we explored previous results that expressed the efficacy of *Panax ginseng* on the development of ALD. The experimental data clearly indicated the beneficial effect of *Panax ginseng* with anti-inflammation, suppression of lipid accumulation in the liver, and antioxidative ability in the prevention and treatment of ALD. These findings would provide novel insights and informative evidence for further trials using the components of *Panax ginseng* for ALD.

## Figures and Tables

**Table 1:** The strategy of literature searching.

PubMed
“alcohol s”[All Fields] OR “alcoholate”[All Fields] OR “alcoholates”[All Fields] OR “alcoholic s”[All Fields] OR “alcoholics”[MeSH Terms] OR “alcoholics”[All Fields] OR “alcoholic”[All Fields] OR “alcoholism”[MeSH Terms] OR “alcoholism”[All Fields] OR “alcoholisms”[All Fields] OR “alcoholism s”[All Fields] OR “alcoholization”[All Fields] OR “alcohols”[MeSH Terms] OR “alcohols”[All Fields] OR “ethanol”[MeSH Terms] OR “ethanol”[All Fields] OR “alcohol”[All Fields]) AND (“liver”[MeSH Terms] OR “liver”[All Fields] OR “livers”[All Fields] OR “liver s”[All Fields]) AND (“ginseng s”[All Fields] OR “panax”[MeSH Terms] OR “panax”[All Fields] OR “ginseng”[All Fields] OR “ginsengs”[All Fields] Texts in all fields were searched. Date of search: 17 January 2023 Result: 113 records were found.
Web of Science
1. (((alcohol) AND (liver)) OR (alcoholic liver disease)) word variations were searched. 2. ((ginseng) OR (panax ginseng)) word variations were searched. 3. #1 AND #2 Texts in all fields were searched. Date of search: 6 February 2023 Result: 41 records were found.
Cochrane Library
1. ((alcohol) OR (alcoholic liver disease)) word variations were searched. 2. ((ginseng) OR (panax ginseng)) word variations were searched. 3. #1 AND #2 Texts in all fields were searched. Date of search: 6 February 2023 Result: 35 records were found.

**Table 2:** General characteristics of included studies.

Major Outcomes	Component	ALD Model	Animal (Sex)	Country	Study
It may prevent liver damage by increasing the resistance of mitochondria to oxidative stress.	Wei Kang Su	Chronic feeding	Rat (Female)	China	[29]
It does not sufficiently reverse the physiological response evoked by long-term ethanol ingestion.	Korean Red Ginseng extract	Single binge, chronic feeding	Rat (Male)	Republic of Korea	[30]
It significantly reduced alcohol-associated steatosis.	Korean Red Ginseng extract	Chronic feeding	Mouse (Male)	Republic of Korea	[31]
It might promote the repression of NF-κB and inhibit the inflammatory reactions in alcoholic hepatitis.	Rg1	Single binge	Mouse (Male)	China	[32]
It may have the potential to treat alcoholic liver disease.	Korean Red Ginseng extract	Chronic feeding	Mouse (N.A.)	Republic of Korea	[33]
The hepatoprotective effect exhibited may be due to its potent antioxidant properties.	Maltol	Multiple binges	Mouse (Male)	China	[34]
It may ameliorate alcoholic fatty liver by suppressing inappropriate lysis of adipose tissue.	Korean Red Ginseng extract	Chronic feeding	Rat (Male)	Republic of Korea	[35]
It is a potent activator of the Nrf2 pathway and could therefore be applied for the prevention of hepatic damage.	Rg1	Chronic feeding	Mouse (Male)	China	[36]
It can markedly increase the levels of ADH and ALDH, decrease EO activity in the liver and decrease the concentration of β-EP and LENK in the brain.	*Panax ginseng*, Hippophae rhamnoides	Single binge	Mouse (Male)	China	[37]
It has a significant protective effect on binge drinking-associated liver injury, and the mechanism is possibly mediated by the partial inhibition of lipopolysaccharide.	*Panax ginseng Meyer*	Single binge	Rat (Male)	China	[38]
It has a potential effect on alcohol-induced liver damage.	Ginseng berry extract	Chronic feeding	Mouse (Male)	Republic of Korea	[39]
It might be a promising candidate treatment agent against alcoholic liver disease.	Ginsenoside Rk3	Chronic feeding	Mouse (Male)	China	[40]
It attenuates alcohol-associated liver injury.	Ginsenoside F2	Multiple binges	Mouse (Male)	Republic of Korea	[26]
It may be used as a potential dietary nutraceutical for alleviating alcohol-associated liver injury.	Fermented *Panax ginseng*, Non-fermented Panax ginseng	Chronic feeding	Mouse (Male)	China	[41]
It significantly reduced the latency of righting reflex and increased the activity.	Fermented *Panax ginseng*, Non-fermented *Panax ginseng*	Single binge	Mouse (Male)	China	[42]
It has a protective role in alcohol-associated hepatitis.	Rg1	Chronic feeding	Mouse (Female)	China	[43]
It may be a promising drug to treat or relieve alcohol-associated liver disease.	Ginsenoside Rc	Chronic feeding	Mouse (Male)	China	[44]
It might be a promising strategy for protection against alcohol-induced liver damage.	Rg1	Chronic feeding	Mouse (Male)	China	[25]

## Data Availability

Published systematic review and PROSPERO (CRD42023389133).

## Supplementary Materials

The following supporting information can be downloaded at https://www.mdpi.com/article/10.3390/antiox12040841/s1. Table S1: Additional characteristics of included studies.
