# Supplementary material for: A Meta-Analysis of Preclinical Studies to Investigate the Effect of Panax ginseng on Alcohol-Associated Liver Disease
Source: Antioxidants (Basel). 2023 Mar 31;12(4):841. doi: 10.3390/antiox12040841 (PMC10135056; doi:10.3390/antiox12040841)
Supplement: Supplementary file 1 [file antioxidants-12-00841-s001.zip › antioxidants-2265292-supplementary.pdf]

antioxidants

MDPI

Table S1. Additional characteristics of included studies.

Study	Diet	Composition of herb	Composition of alcohol	Route and times	Dose
[29]	N.A.	*Radix Ginseng*/*Radix Quinquefolium* (1:20, wt/wt), *Radix Ophiopogonis*, and *Fructus Schisandrae Chinensis* in a weight-to-weight ratio of 1:3:2	20% ethanol	Oral, once daily	125 mg/kg,<br>375 mg/kg
[30]	AIN-93G	Roots of six-year-old Korean Red Ginseng dissolved directly in 5% diluted ethanol (1.0% as final concentration)	35% ethanol	Oral, once daily	47 mg/kg
[31]	Standard chow diet	Undiluted solution of Korean Red Ginseng containing Rg1 (2.481), Rb1 (5.481), Rg3 (0.197), Re (2.975), Rc (2.248), Rb2 (2.175), and Rb (0.566)	10% ethanol	Oral, 5 times a week	200 mg/kg
[32]	Standard chow diet	Rg1 (purity 98%) in powder form	56% ethanol (Hongxing Brewery)	Oral, once	10 mg/kg,<br>20 mg/kg,<br>40 mg/kg
[33]	Lieber-DeCarli liquid diet	Roots of six-year-old *Panax Ginseng Meyer* extracted six times with water at 87° C for 12 h	20% ethanol	Oral, once daily	250 mg/kg,<br>500 mg/kg,<br>250 mg/kg,<br>500 mg/kg
[34]	N.A.	Roots of *Panax Ginseng Meyer*	50% ethanol	Oral, once daily	25 mg/kg<br>50 mg/kg
[35]	Dextrin-maltose diet	Roots of six-year-old *Panax Ginseng Meyer* extracted with hot water and concentration	5% ethanol	Oral, once daily	125 mg/kg<br>250 mg/kg
[36]	Dextrin-maltose diet	Rg1 in powder form	36% ethanol	Oral, once daily	10 mg/kg<br>20 mg/kg<br>40 mg/kg
[37]	N.A.	*Panax Ginseng Meyer* 100g and *Hippophae rhamnoides* 400 g extracted with distilled water at 90° C for 6 h	Pure ethanol	Oral, once	100 mg/kg<br>200 mg/kg<br>400 mg/kg
[38]	Standard chow diet	Roots of *Panax Ginseng Meyer* extracted with enzymatic hydrolysis	50% ethanol	Oral, once	62.5 mg/kg<br>125 mg/kg<br>250 mg/kg<br>500 mg/kg
[39]	N.A.	Berries of six-year-old *Panax Ginseng Meyer* extracted with enzymatic hydrolysis	25% ethanol	Oral, once daily	0.5 mg/mouse<br>2 mg/mouse<br>5 mg/mouse
[40]	N.A.	Standard ginsenoside Rk3 (purity 99.0%) dissolved in 50% deionized water and 50% polyethylene glycol 400	50% ethanol	Oral, once daily	25 mg/kg<br>50 mg/kg
[26]	N.A.	Ginsenoside F2 purified using recycling preparative high-performance liquid chromatography	40% ethanol	Oral, once daily	50 mg/kg
[41]	Lieber-DeCarli liquid diet	Five-year-old *Panax Ginseng Meyer* fermented with *Lactobacillus fermentum* KP-3	Ethanol	Oral, once daily	390 mg/kg
[42]	N.A.	Sprouts of 30-days-old *Panax Ginseng Meyer* fermented with *Lactobacillus plantarum* and *Lactobacillus brevis*	Ethanol	Oral, once	10 mg/kg<br>2.5 ml/kg<br>10 ml/kg
[43]	Standard chow diet	Ginsenoside Rg1 (purity 98.5%)	5% ethanol	Oral, once daily	40 mg/kg
[44]	Dextrin-maltose diet	Ginsenoside Rc (purity 98%)	Ethanol	Oral, once daily	5 mg/kg<br>10 mg/kg<br>20 mg/kg
[25]	N.A.	Ginsenoside Rg1 (purity 98%)	Ethanol	Oral, once daily	10 mg/kg<br>40 mg/kg
